# Assessment of antimalarial drug resistant markers in asymptomatic *Plasmodium falciparum* infections after 4 years of indoor residual spraying in Northern Ghana

**DOI:** 10.1371/journal.pone.0233478

**Published:** 2020-12-07

**Authors:** James L. Myers-Hansen, Benjamin Abuaku, Muyiwa K. Oyebola, Benedicta A. Mensah, Collins Ahorlu, Michael D. Wilson, Gordon Awandare, Kwadwo A. Koram, Alfred Amambua Ngwa, Anita Ghansah

**Affiliations:** 1 Noguchi Memorial Institute for Medical Research, University of Ghana, Legon, Ghana; 2 West African Center for Cell Biology of Infectious Pathogens, University of Ghana, Legon, Ghana; 3 Medical Research Council Unit The Gambia at LSHTM, Banjul, Gambia; Instituto Rene Rachou, BRAZIL

## Abstract

**Background:**

Drug resistance remains a concern for malaria control and elimination. The effect of interventions on its prevalence needs to be monitored to pre-empt further selection. We assessed the prevalence of *Plasmodium falciparum* gene mutations associated with resistance to the antimalarial drugs: sulfadoxine-pyrimethamine (SP), chloroquine (CQ) and artemisinin combination therapy (ACTs) after the scale-up of a vector control activity that reduced transmission.

**Methods:**

A total of 400 *P*. *falciparum* isolates from children under five years were genotyped for seventeen single nucleotide polymorphisms (SNPs) in *pfcrt*, *pfmdr1*, *pfdhfr*, *pfdhps* and *pfk13* genes using polymerase chain reaction (PCR) and high resolution melting (HRM) analysis. These included 80 isolates, each randomly selected from cross-sectional surveys of asymptomatic infections across 2010 (baseline), 2011, 2012, 2013 (midline: post-IRS) and 2014 (endline: post-IRS) during the peak transmission season, when IRS intervention was rolled out in Bunkpurugu Yunyoo (BY) District, Ghana. The proportions of isolates with drug resistant alleles were assessed over this period.

**Results:**

There were significant decreases in the prevalence of *pfdhfr*- I_51_R_59_N_108_ haplotype from 2010 to 2014, while the decline in *pfdhfr/pfdhps-* I_51_R_59_N_108_G_437_ during the same period was not significant. The prevalence of lumefantrine (LM), mefloquine (MQ) and amodiaquine (AQ) resistance-associated haplotypes *pfmdr1*-N_86_F_184_D_1246_ and *pfmdr1*-Y_86_Y_184_Y_1246_ showed decreasing trends (z = -2.86, P = 0.004 and z = -2.71, P = 0.007, respectively). Each of *pfcrt-*T76 and *pfmdr1*-Y86 mutant alleles also showed a declining trend in the asymptomatic reservoir, after the IRS rollout in 2014 (z = -2.87, P = 0.004 and z = -2.65, P = 0.008, respectively). Similarly, Pyrimethamine resistance mediating polymorphisms *pfdhfr*-N108, *pfdhfr*-I51 and *pfdhfr*-R59 also declined (z = -2.03, P = 0.042, z = -3.54, P<0.001 and z = -4.63, P<0.001, respectively), but not the sulphadoxine resistance mediating *pfdhps*-G437 and *pfdhps*-F436 (z = -0.36, P = 0.715 and z = 0.41, P = 0.684, respectively). No mutant *pfk13*-Y580 were detected during the study period.

**Conclusion:**

The study demonstrated declining trends in the prevalence of drug resistant mutations in asymptomatic *P*. *falciparum* infections following transmission reduction after an enhanced IRS intervention in Northern Ghana.

## Introduction

In spite of all the gains made in global malaria control over the past fifteen years, progress stalled between 2015–2018 [1], resulting in 228 million cases and 405, 000 deaths in 2018 alone [1]. The disease burden is highest in sub-Saharan Africa where children under five years are the most affected [1] and *Plasmodium falciparum* resistance to antimalarial treatment continues to threaten the efforts at elimination of malaria [2–5].

In Ghana, the disease is the leading cause of morbidity and mortality and is transmitted perennially in the southern (coastal) and middle (forest) belts, and seasonally in the northern (savanna) belt [6]. In 2015, the country recorded approximately 10 million suspected malaria cases with 31% in children under five years. An estimated 2100 malaria-related deaths were also recorded in 2015 of which children under five formed about 50% [7]. The primary interventions for malaria control in Ghana are: early diagnosis with prompt and effective treatment using artemisinin based combination therapies (ACTs); scaling-up of vector control measures that emphasize, universal Insecticide-treated nets (ITN) coverage; targeted Indoor Residual Spraying (IRS) in selected areas; seasonal malaria chemoprevention (SMC); and Intermittent Preventive Treatment in pregnancy (IPTp). As in other sub-Saharan African countries, chloroquine (CQ) use in Ghana was discontinued and replaced with ACTs, due to antimalarial resistance. Specifically, artesunate-amodiaquine (ASAQ) was introduced in 2005 and subsequently artemether-lumefantrine (AL) and dihydroartemisinin-piperaquine (DHAP) were included as additional first-line treatments for uncomplicated malaria in 2007 and 2009, respectively [8]. Sulphadoxine Pyrimethamine (SP) was deployed as prophylaxis in IPTp in 2004 and SP+AQ for SMC among children under five years in the northern regions of the country since 2015. The 2014 Ghana Demographic and Health Survey (DHS) [9] and the 2016 Ghana Malaria Indicator Survey (MIS) [10] reported the use of ACTs by 78.2% and 53% of Ghanaian children under age 5 years, respectively. ITN use has increased from 3% in 2003 to over 60% of children by 2011, following mass free distribution campaigns in many parts of the country [11].

With support from the U.S President’s Malaria Initiative (PMI), the U.S Centers for Disease Control (CDC) and ABT Associates, the Ghana Health Service (GHS) in collaboration with the Noguchi Memorial Institute for Medical Research (NMIMR) in 2010 implemented extensive IRS surveys in the Bunkpurugu Yunyoo (BY) District of the Northern Region of Ghana. The aim was to significantly reduce malaria transmission intensity and parasitaemia, as part of the country’s malaria control strategies. As a result of the program, the prevalence of parasitaemia in children under age five declined from 52.4% in 2010 to 22.2% in 2014 [12] and entomological inoculation rate (ie. transmission intensity) dropped from 0.350 to 0.010 infectious bites/person/night during the same period (Dadzie et al., manuscript under review).

Despite these achievements, Ghana was one of the 12 most malaria-burdened African countries according to the WHO, representing 3% of the global malaria burden with an increase in malaria cases in 2018 compared with 2017 [1]. This raises questions about the sustainability of transmission reduction as a result of these vector control interventions and calls for a renewed drive in malaria control.

Treatment, prophylaxis and anti-vector interventions reduce parasite prevalence, transmission, morbidity, and mortality, but how the interactions between these interventions and the resultant change in epidemiology affect the prevalence of drug resistant markers is not fully understood. There remains a reservoir of asymptomatic *P*. *falciparum* infection in circulation predominantly in older children and adults, that sustains transmission and thus contributes to the spread of antimalarial resistance even after highly successful vector control measures. This asymptomatic reservoir represents infections in individuals with varying parasite densities, but with no fever or other acute symptoms that would warrant them to seek treatment [13, 14]. A proportion of asymptomatic parasitaemia is submicroscopic and only identifiable with molecular methods, the other is detectable using microscopy and rapid diagnostic tests (RDT) [14,15]. The ability to detect and manage these infections thus remains vital to the success of elimination. Ascertaining how the remaining reservoir would respond to anti-malarial therapy after an intervention has reduced transmission intensity would also have implications for future malaria control activities in a locale.

While some studies have found an association between transmission reduction and a decline in the drug resistance evolution [16–18], others have reported a reverse association [19,20] and some others have found no clear association [21–23]. Other studies have also found a mixed association [24]. Transmission reduction may indeed suppress the development of drug resistance [16–18], however, at very low transmission intensity, it may likely increase the evolution of drug resistance [24]. At very low transmission intensities, parasite populations tend to inbreed significantly and sexual recombination is reduced. As such, drug resistant gene combinations in the population stabilize and rapidly increase in frequency [20,25].

Resistance to antimalarial drugs is attributed to single nucleotide polymorphisms (SNPs) in different *P*. *falciparum* genes, including: the chloroquine resistance transporter *(pfcrt)* gene on chromosome 7 (associated with chloroquine resistance) [26,27]; the multidrug resistant (*pfmdr1)* gene on chromosome 5 (associated with chloroquine, mefloquine, amodiaquine, lumefantrine and artemisinins resistance) [28–30]; the dihydropteroate synthetase (*pfdhps)* gene on chromosome 8 (associated with sulphadoxine resistance) [31,32]; the dihydrofolate reductase *(pfdhfr)* gene on chromosome 4 (associated with pyrimethamine resistance) [33,34]; and the Kelch 13 (*pfk13)* gene on chromosome 13 (associated with artemisinin resistance) [35–37]. The use of these molecular markers to detect and monitor drug resistant parasites comprise one of the most valuable methods in assessing antimalarial drug efficacy.

In this study, we assessed temporal trends in the prevalence of mutations in *pfcrt*, *pfmdr1*, *pfdhps*, *pfdhfr* and *pfk13* genes in *P*. *falciparum* isolates from cross-sectional surveys of the most malaria vulnerable group during the peak transmission season in the BY district. Multidrug resistance and linkage between drug resistance genes were also evaluated with clonal infections. Ultimately, this study will provide information that will guide subsequent targeted malaria control strategies in Ghana.

## Materials and methods

### Study site and samples

**Fig 1:**
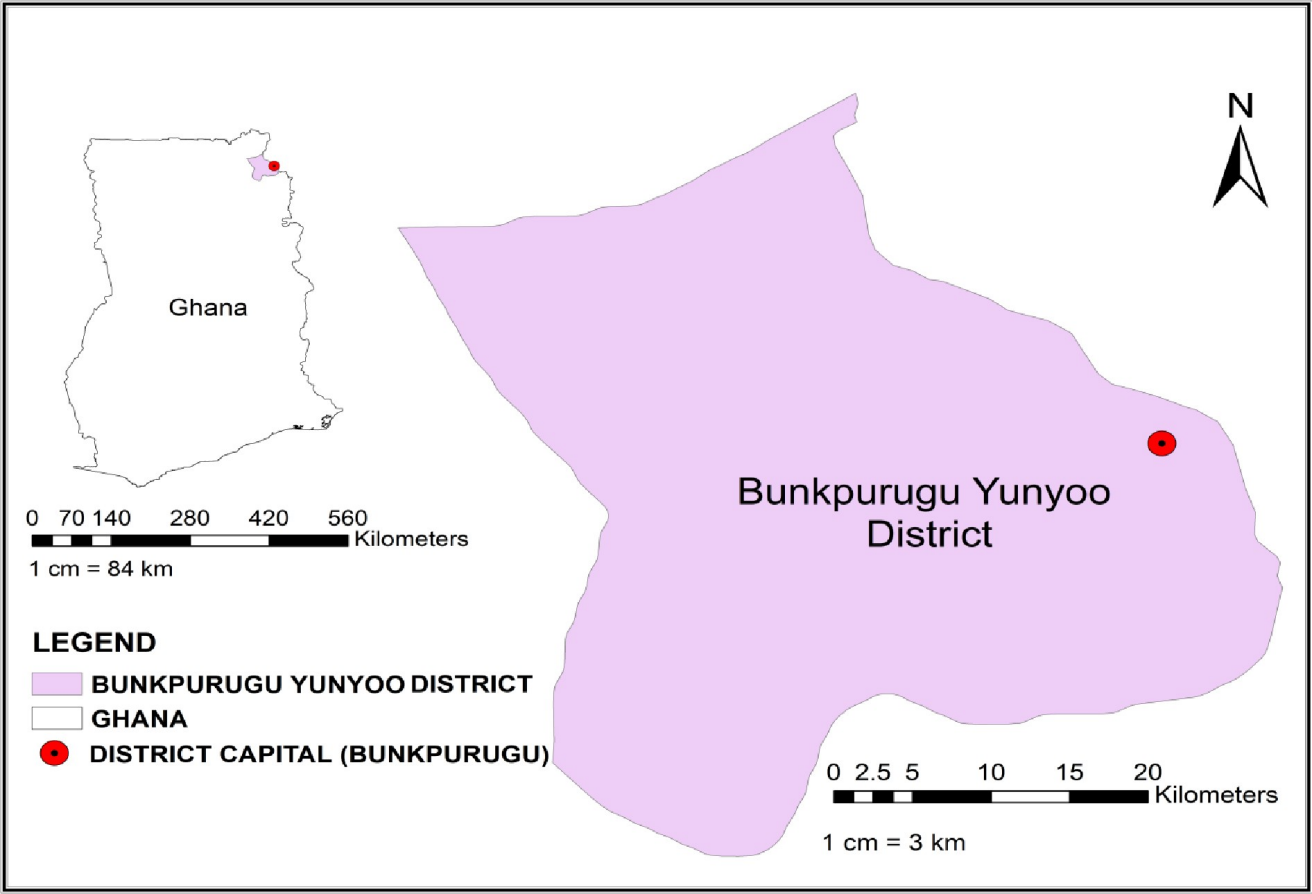
Map of Ghana showing location of Bunkpurugu Yunyoo District.

This study was part of a yearly IRS intervention carried out between 2010 and 2014 in Bunkpurugu Yunyoo District of the Northern Region of Ghana [Fig 1], where malaria transmission is high and seasonal. The comprehensive study design and features for the IRS intervention have been described elsewhere [12]. In the course of the IRS intervention, cross-sectional surveys were conducted in 50 communities in March/April (end of the dry season) and October/November (end of the rainy season) to determine the impact of IRS on *P*. *falciparum* parasitaemia and severe anaemia in children under the age of five. For each survey, Dried blood blots (DBS) (one per child) were obtained from all study participants following rapid malaria testing [CareStart^TM^Malaria HRP2/pLDH (pf/PAN) Combo (AccessBio, New Jersey, USA)], light microscopy, and anaemia testing (Hemocue AB, Ӓngelholm, Sweden). Parasite quantification and species identification were done using thick and thin smears, respectively. Briefly, parasites were quantified against 200 white blood cells (WBC) as counts per microliter of blood, assuming 8000 WBC per microliter of blood. Two senior microscopists independently read all blood slides and a third senior microscopist read the discordant slides pertaining to species identification and sexual/asexual parasite presence. The third microscopist’s reading was considered the decider for all discordant slides [12]. Mixed infections of *P*. *falciparum* with *P*. *malariae* accounted for 1.5–4.9% and *P*. *falciparum* with *P*. *ovale* was 0.2–2.3% [12], and these were excluded from our analysis. Information based on demographic, clinical, parasitological and entomological assessments were also obtained. For this study, we generated random numbers for sample selection from the sample list captured in an excel spreadsheet. We randomly selected 80 *P*. *falciparum* mono-infected smear-positive blood samples each from 2010 (baseline), 2011, 2012, 2013 (midline: post-IRS) and 2014 (endline: post-IRS), from the peak transmission (end of rainy season) season and the same sub-districts were used for downstream analysis.

### Ethical statement

All aspects of this study were approved by the Institutional Review Board of the Noguchi Memorial Institute for Medical Research (NMIMR IRB; FWA Number: 00001824), Accra, Ghana (Study Number: 009/10-11). Details of the study were explained in local dialect to the legal parents or guardians of the participants and each signed a written consent form.

### SNP genotyping of drug resistance markers

Genomic DNA was extracted from slide positive DBSs using QiaAmp DNA Blood Mini Kit (Qiagen GMBH, Hilden, Germany), following the manufacturer’s protocol and stored at -20˚C before use. Asymmetrical PCR and high resolution melting (HRM) curve analysis were carried out to genotype alleles in *pfcrt* (C72S, M74I, N75E, K76T), *pfmdr1* (N86Y, Y184F, D1246Y), *pfdhps* (S436F, A437G, K540E, A581G, A613S), *pfdhfr* (N51I, C59R, S108N, I164L) and *pfk13* C580Y that are associated with antimalarial drug resistance. All PCRs were performed in a final volume of 5μL containing 1X LightScanner Master mix (BioFire Defense, Salt Lake City, UT. USA), 0.5μM excess primer (reverse), 0.1μM limiting primer (forward), 0.4 μM 3’-blocked probe and 0.01–10 ng/μl template DNA. Previously reported primer sets and cycling conditions were used [38,39]. Following PCR, the amplified products were analysed using HRM from 40˚C to 90˚C on the Roche LightCycler96 instrument (Roche Diagnostics, Germany). The LightCycler 96 SW 1.1 software was used for the analyses of melting profiles and allele calling based on the changes in fluorescence. Purified genomic DNA from laboratory-adapted *P*. *falciparum* strains (3D7, Dd2, HB3, TM90, 1241) with known genotype profiles for wildtype and mutant alleles were used as standards to discriminate sensitive and resistant alleles in samples.

### Data interpretation

Melting curves/peaks representative of each allele was used to ascertain the SNP genotype of each sample. For a sample containing both wildtype and mutant alleles for a particular SNP, the melting profile showed two distinct melting peaks/curves representative of each allele.

The *pfcrt*-C72S, M74I, N75E, K76T and *pfmdr1*-N86Y, Y184F, D1246Y SNPs were analysed individually and defined as either wildtype, “infection with no mutation detected” or mutant, “infection with mutation detected”. Similarly, the *pfdhfr*-N51I, C59R, S108N, I164L; *pfdhps*-S436F, A437G, K540E, A581G, A613S; and *pfk13*-C580Y point mutations were also classified as wildtype or mutant.

Single gene haplotypes were categorized as: wildtype- “infection with no mutation detected” or either single, double, triple, quadruple, quintuple, sextuple and septuple mutants for infections with 1, 2, 3, 4, 5, 6 and 7 mutant alleles respectively. Thus *pfcrt* (C72S, M74I, N75E, K76T) haplotypes were either wildtype or triple, whiles *pfmdr1* (N86Y, Y184F, D1246Y) haplotypes were wildtype, single, double or triple. Similarly, *pfdhfr* (N51I, C59R, S108N, I164L) haplotypes were defined as wildtype, single, double, triple or quadruple, whiles that of the *pfdhps* (S436F, A437G, K540E, A581G, A613S) were categorized as wildtype, single, double, triple, quadruple or quintuple. Multi-gene haplotypes were constructed for *pfcrt/pfmdr1* (C72S, M74I, N75E, K76T, N86Y, Y184F, D1246Y), *pfdhfr/pfdhps* (N51I, C59R, S108N, A437G, K540E, A581G) and *pfcrt/pfmdr1/pfdhfr/pfdhps* (K76T, N86Y, N51I, C59R, S108N, A437G, K540E).

The prevalence of mutations, were determined as the proportion of samples carrying the target non-wildtype variant among the total number of successfully analysed samples. In estimating the prevalence of mutations and for haplotype analysis, all infections with mixed alleles (wildtype and mutant) were excluded.

### Data analysis

The demographic data of study participants (proportion of males and females, mean age and mean parasitaemia) and prevalence of alleles in *pfcrt* (C72S, M74I, N75E, K76T), *pfmdr1* (N86Y, Y184F, D1246Y), *pfdhps* (S436F, A437G, K540E, A581G, A613S), *pfdhfr* (N51I, C59R, S108N, I164L) and *pfk13* C580Y were consolidated for each survey year. Differences in mean age and parasitaemia among the survey years were analysed using ANOVA. The Chi-square (χ^2^) and Fisher exact tests were used to determine the significance of observed differences in the proportion of sexes (male and female) and the prevalence of key drug resistance alleles. The trends in the prevalence of drug resistance alleles over the study period, from 2010 to 2014 were analysed using the Cuzick’s (nptrend) test. Statistical analysis was performed using STATA software package version 12 (College Station, TX: StataCorp LP. USA). P values <0.05 were considered to indicate statistical significance.

## Results

### Study population

The distribution of sex and age of study participants were comparable across the study period (χ^2^ = 2.66, P = 0.616 and F = 0.09, P = 0.986, respectively). The proportion of males and females were 53.8 and 46.2% for 2010, 47.5 and 52.5% for 2011, 42.5 and 57.5% for 2012, 51.2 and 48.8% for 2013, and 52.5 and 47.5% for 2014. The mean ages (in months) were 32.74, 33.56, 33.26, 32.64 and 32.20 for 2010 to 2014 respectively. The geometric mean parasitaemia (in parasites/μL) however differed significantly across the study period (6077, 15099, 2602, 4317 and 3563, F = 4.74, P = 0.001), with an overall decline of 58.6% (Table 1).

**Table 1:** Background characteristics of study participants in Bunkpurugu Yunyoo District across survey years.

Characteristic	2010	2011	2012	2013	2014	P-value
**Gender [% (n/N)]**						
**Male**	53.8 (43/80)	47.5 (38/80)	42.5 (34/80)	51.3 (41/80)	52.5 (42/80)	0.616
**Female**	46.2 (37/80)	52.5 (42/80)	57.5 (46/80)	48.7 (39/80)	47.5 (38/80)	
**Age [mean ± SD] (months)**	32.74 ± 16.56	33.56 ± 16.08	33.26 ± 16.42	32.64 ± 16.43	32.20 ± 15.61	0.986
**GMPD [Range] (parasites/μL)**	6077 [520–85960]	15099 [800–294400]	2602 [120–232673]	4317 [197–489216]	3563 [123–133714]	0.001

GMPD, geometric mean parasite density (in parasites/μL). Statistical analysis performed using Pearson’s chi-square and ANOVA tests.

### Genotyping success

For the 400 (80 each for 5 years) asymptomatic *P*. *falciparum* isolates selected from 2010 to 2014, the genotyping success ranged between 73.8% (59/80) to 93.8% (75/80) for *pfcrt*; 86.3% (69/80) to 97.5% (78/80) for *pfmdr1*; 85.0% (68/80) to 100% (80/80) for *pfdhfr;* 86.3% (69/80) to 98.8% (79/80) for *pfdhps*; 70.0% (56/80) to 91.3% (73/80) for *pfk13* as shown in S1 Table. Samples that were not successfully genotyped for each of the five genes did not differ in gender (P = 0.225 to 0.728) and age (P = 0.1439 to 0.9924) compared to those that were successfully genotyped. The success of genotyping was independent of parasitaemia, as there was no significant difference in geometric mean parasitaemia between successfully genotyped and failed samples for most of the genes except for *pfcrt* and *pfk13* genes [*pfcrt*:1318 and 6479 parasites/μL, *pfk13*: 2091 and 6214 parasites/μL for nonsuccessful and successful respectively, F = 7.11, P = 0.008 and F = 5.95, P = 0.015)].

### Prevalence and temporal trends of key *pfcrt*, *pfmdr1*, *pfdhf*, *pfdhps*, *pfk13* drug resistant point mutations

**Fig 2:**
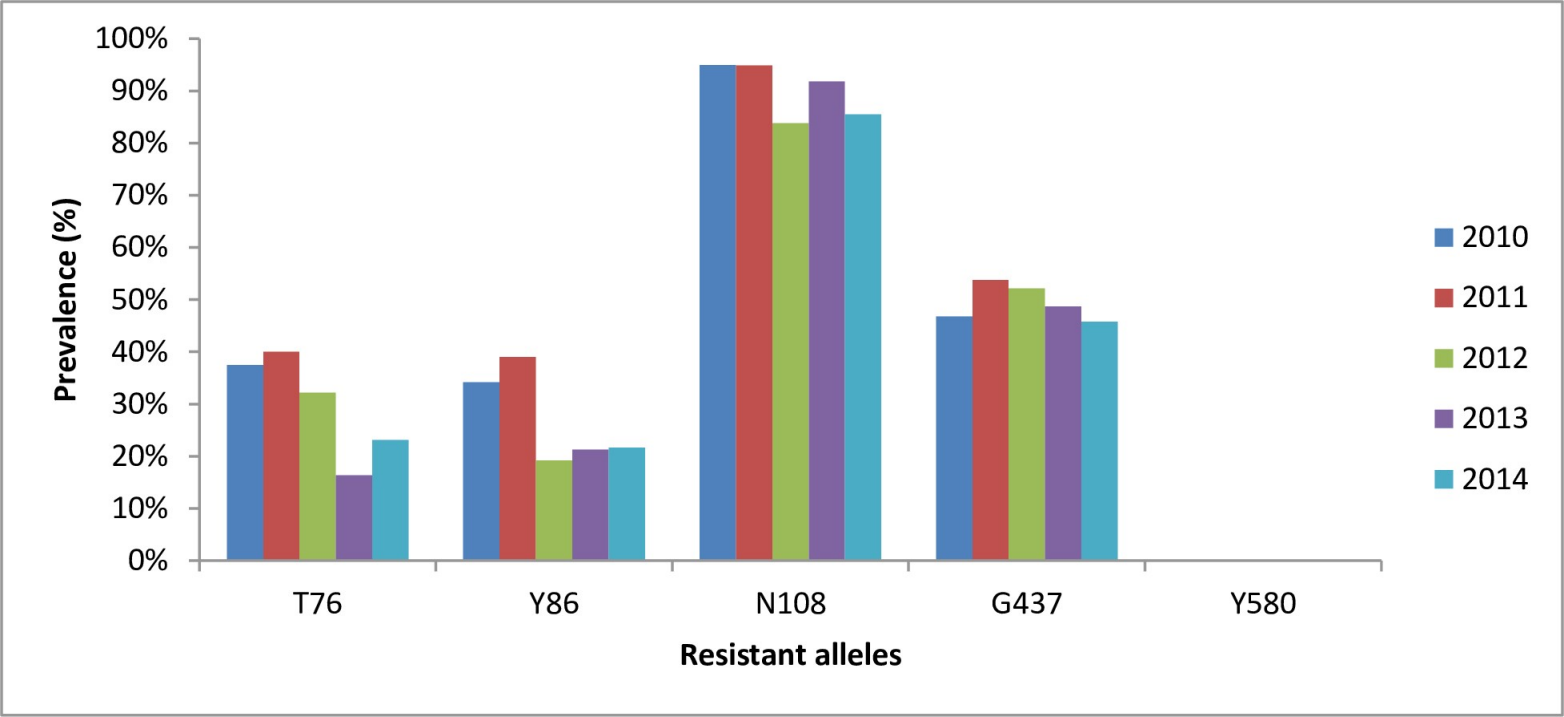
Trends in prevalence of *pfcrt-*T76, *pfmdr1-*Y86, *pfdhfr-*N108, *pfdhps-*G437 and *pfk13*-Y580 mutant alleles in natural *P*. *falciparum* populations in the BY district of Northern Ghana, at baseline (2010), during (2011–2013) and after enhanced vector control intervention with IRS (2014). Statistical analysis performed using Cuzick’s test for trends.

The prevalence of key chloroquine resistant alleles: *pfcrt*-T76 and *pfmdr1*-Y86 were lower than the wildtype alleles and fluctuated during the study period: 37.5% and 34.2% in 2010, 40.0% and 39.0% in 2011, 32.2% and 19.2% in 2012, 16.4% and 21.3% in 2013, and 23.1% and 21.7% in 2014, respectively (χ^2^ = 12.76, P = 0.013 and χ^2^ = 11.91, P = 0.018). There was a significant decline in the prevalence of these alleles from baseline, during and post-IRS intervention (z = -2.87, P = 0.004 and z = -2.65, P = 0.008, respectively) [Fig 2].

Similarly, the prevalence of *pfdhfr*-N108 and *pfdhps*-G437 mutations wavered during the period. For 2010, 2011, 2012, 2013 and 2014 these were 95.0% and 46.8%, 94.9% and 53.8%, 83.8% and 52.2%, 91.8% and 48.7%, and 85.5% and 45.8% respectively (χ^2^ = 9.37, P = 0.057 and χ^2^ = 1.42, P = 0.841). Only the pyrimethamine resistance mediating mutant *pfdhfr*-N108 showed a trend in decline in prevalence after the intervention (z = -2.03, P = 0.042) [Fig 2]. The core Kelch-13 artemisinin resistant associated allele (*pfk13*-Y580) was not found throughout the survey years.

### Prevalence and temporal trends of other *pfcrt*, *pfmdr1*, *pfdhfr* and *pfdhps* drug resistant point mutations

No mutant *pfcrt*-S72 was identified across the survey years, while *pfcrt*-I74 and *pfcrt*-E75 mutations were detected in 37.5% of the asymptomatic *P*. *falciparum* isolates surveyed for 2010, after which their prevalence declined (z = -2.87, P = 0.004) following subsequent years of survey: thus from 40% of isolates in 2011 to 32.2% in 2012 and then 16.4% and 23.1% isolates in 2013 and 2014 respectively. The prevalence in *pfmdr1* mutant alleles, *pfmdr1-* F184 and *pfmdr1*-Y1246 also showed a trend in decline across the survey period, (z = -3.47, P = 0.001 and z = -2.11, P = 0.035, respectively).

The prevalence of the pyrimethamine resistance mediating polymorphisms in *pfdhfr* gene was generally higher than the sulphadoxine resistance mediating polymorphisms *in pfdhps*. There was a significant decline in the trend of *pfdhfr*-I51 and *pfdhfr*-R59 after the intense IRS was rolled out in BY (z = -3.54, P<0.001 and z = -4.63, P<0.001, respectively). There was no *pfdhfr*-L164 mutation found throughout the years of survey. The prevalence of the least abundant mutant *pfdhps*-F436 and G581 non-significantly increased from 6.3% to 9.7% (z = 0.41, P = 0.684) and 0.0% to 2.8% (z = 1.52, P = 0.129), respectively between baseline and post-intervention surveys. Within that same period, the least frequent *pfdhps*-S613 mutant decreased non-significantly in prevalence from 29.1% in 2010 to 15.3% in 2014, (z = -1.86, P = 0.063).

The mutant *pfdhps*-E540 was however absent across all time points. The trends in the prevalence of the *pfcrt*, *pfmdr1*, *pfdhfr and pfdhps* mutations are summarized in Table 2 below.

**Table 2:** Trends in the prevalence of *pfcrt*, *pfmdr1*, *pfdhfr* and *pfdhps* alleles across survey years.

Gene	Mutation	Base change	Amino acid	2010% (n/N)	2011% (n/N)	2012% (n/N)	2013% (n/N)	2014% (n/N)	P-value
***Pfcrt***	C72S	T	C	100 (75/75)	100 (73/73)	100 (59/59)	100 (70/70)	100 (66/66)	
		A	**S**	0.0 (0/75)	0.0 (0/73)	0.0 (0/59)	0.0 (0/70)	0.0 (0/66)	
	M74I	A	M	62.5 (45/72)	60.0 (42/70)	67.8 (40/59)	83.6 (56/67)	76.9 (50/65)	0.004
		G	**I**	37.5 (27/72)	40.0 (28/70)	32.2 (19/59)	16.4 (11/67)	23.1 (15/65)	
	N75E	T	N	62.5 (45/72)	60.0 (42/70)	67.8 (40/59)	83.6 (56/67)	76.9 (50/65)	0.004
		A	**E**	37.5 (27/72)	40.0 (28/70)	32.2 (19/59)	16.4 (11/67)	23.1 (15/65)	
	K76T	A	K	62.5 (45/72)	60.0 (42/70)	67.8 (40/59)	83.6 (56/67)	76.9 (50/65)	0.004
		C	**T**	37.5 (27/72)	40.0 (28/70)	32.2 (19/59)	16.4 (11/67)	23.1 (15/65)	
***Pfmdr1***	N86Y	A	N	65.8 (48/73)	61.0 (47/77)	80.8 (59/73)	78.7 (59/75)	78.3 (54/69)	0.008
		T	**Y**	34.2 (25/73)	39.0 (30/77)	19.2 (14/73)	21.3 (16/75)	21.7 (15/69)	
	Y184F	A	Y	9.7 (7/72)	13.7 (10/73)	13.9 (10/72)	21.7 (15/69)	29.8 (20/67)	0.001
		T	**F**	90.3 (65/72)	86.3 (63/73)	86.1 (62/72)	78.3 (54/69)	70.2 (47/67)	
	D1246Y	G	D	90.4 (66/73)	94.9 (74/78)	95.9 (70/73)	96.0 (72/75)	98.6 (68/69)	0.035
		T	**Y**	9.6 (7/73)	5.1 (4/78)	4.1 (3/73)	4.0 (3/75)	1.4 (1/69)	
***Pfdhfr***	N51I	A	N	32.5 (25/77)	26.7 (20/75)	44.8 (30/67)	47.9 (34/71)	55.2 (37/67)	<0.001
		T	**I**	67.5 (52/77)	73.3 (55/75)	55.2 (37/67)	52.1 (37/71)	44.8 (30/67)	
	C59R	T	C	20.8 (16/77)	18.7 (14/75)	41.8 (28/67)	42.3 (30/71)	50.7 (34/67)	<0.001
		C	**R**	79.2 (61/77)	81.3 (61/75)	58.2 (39/67)	57.7 (41/71)	49.3 (33/67)	
	S108N	G	S	5.0 (4/80)	5.1 (4/79)	16.2 (11/68)	8.2 (6/73)	14.5 (10/69)	0.042
		A	**N**	95.0 (76/80)	94.9 (75/79)	83.8 (57/68)	91.8 (67/73)	85.5 (59/69)	
	I164L	A	I	100 (80/80)	100 (79/79)	100 (68/68)	100 (73/73)	100 (69/69)	
		T	**L**	0.0 (0/80)	0.0 (0/79)	0.0 (0/68)	0.0 (0/73)	0.0 (0/69)	
***Pfdhps***	S436F	C	S	93.7 (74/79)	88.4 (69/78)	92.8 (64/69)	91.0 (71/78)	90.3 (65/72)	0.684
		T	**F**	6.3 (5/79)	11.6 (9/78)	7.2 (5/69)	9.0 (7/78)	9.7 (7/72)	
	A437G	C	A	53.2 (42/79)	46.2 (36/78)	47.8 (33/69)	51.3 (40/78)	54.2 (39/72)	0.715
		G	**G**	46.8 (37/79)	53.8 (42/78)	52.2 (36/69)	48.7 (38/78)	45.8 (33/72)	
	K540E	A	K	100 (79/79)	100 (78/78)	100 (69/69)	100 (78/78)	100 (72/72)	
		G	**E**	0.0 (0/79)	0.0 (0/78)	0.0 (0/69)	0.0 (0/78)	0.0 (0/72)	
	A581G	C	A	100 (79/79)	98.7 (77/78)	92.7 (64/69)	94.9 (74/78)	97.2 (70/72)	0.129
		G	**G**	0.0 (0/79)	1.3 (1/78)	7.3 (5/69)	5.1 (4/78)	2.8 (2/72)	
	A613S	G	A	70.9 (56/79)	87.2 (68/78)	88.4 (61/69)	84.6 (66/78)	84.7 (61/72)	0.063
		T	**S**	29.1 (23/79)	12.8 (10/78)	11.6 (8/69)	15.4 (12/78)	15.3 (11/72)	
***Pfk13***	C580Y	G	C	100 (70/70)	100 (65/65)	100 (56/56)	100 (68/68)	100 (73/73)	
		A	**Y**	0.0 (0/70)	0.0 (0/65)	0.0 (0/56)	0.0 (0/68)	0.0 (0/73)	

Note. Wild type amino acids are depicted in normal font, while mutant amino acids are in bold and underlined. Statistical analysis performed using Cuzick’s test for trends.

### *Pfcrt* and *pfmdr1* haplotype frequencies and trends across survey years

**Fig 3:**
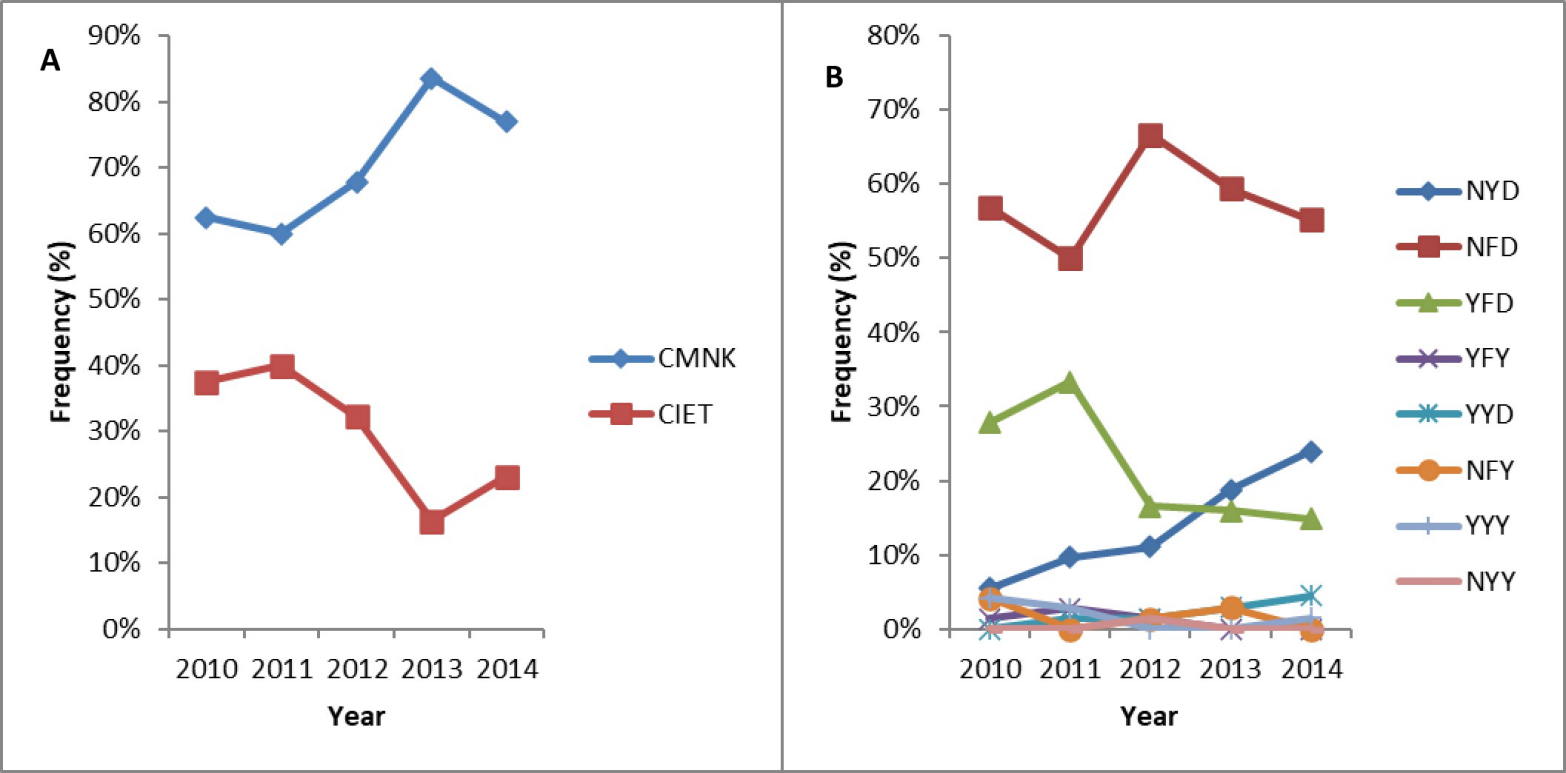
Trends in the frequency of *pfcrt* and *pfmdr1* haplotypes at baseline, during and after intervention. **(A)** *pfcrt*, **(B)** *pfmdr1*. Statistical analysis performed using Cuzick’s test for trends.

To determine the effect of transmission reduction on the observed *pfcrt* and *pfmdr1* haplotype frequencies and their trends across survey years, combinations of alleles per isolate were considered as haplotypes across loci and frequencies compared across temporal populations. Overall, two unique *pfcrt* haplotypes were detected across the study period and in each year of survey, with the wildtype C_72_M_74_N_75_K_76_ being the most abundant haplotype, ranged from 60.0–83.6%. The predominant African/South East Asian triple mutant, *pfcrt*- C_72_I_74_E_75_T_76_ haplotype associated with chloroquine resistance showed a statistically significant trend in decline (z = -2.87, P = 0.004), between baseline and post-intervention. On the other hand, eight unique *pfmdr1* haplotypes were found across the survey time points, with six in 2010 and 2011, seven in 2012, five in 2013 and 2014. The *pfmdr1* single mutant N_86_F_184_D_1246_ haplotype that is associated with low lumefantrine response persisted from baseline, during and post-IRS intervention and the frequency ranged from 50.0–66.7%. There was a decline in the frequency of the single and double *pfmdr1* mutant haplotypes N_86_F_184_D_1246_ and Y_86_Y_184_Y_1246_ associated with lumefantrine and amodiaquine resistance, respectively (z = -2.86, P = 0.004 and z = -2.71, P = 0.007), with fluctuating frequencies between baseline and post-intervention surveys. The temporal trends in *pfcrt* and *pfmdr1* haplotype frequencies across survey years are presented in Fig 3.

### *Pfdhfr* and *pfdhps* haplotype frequencies and trends across survey years

**Fig 4:**
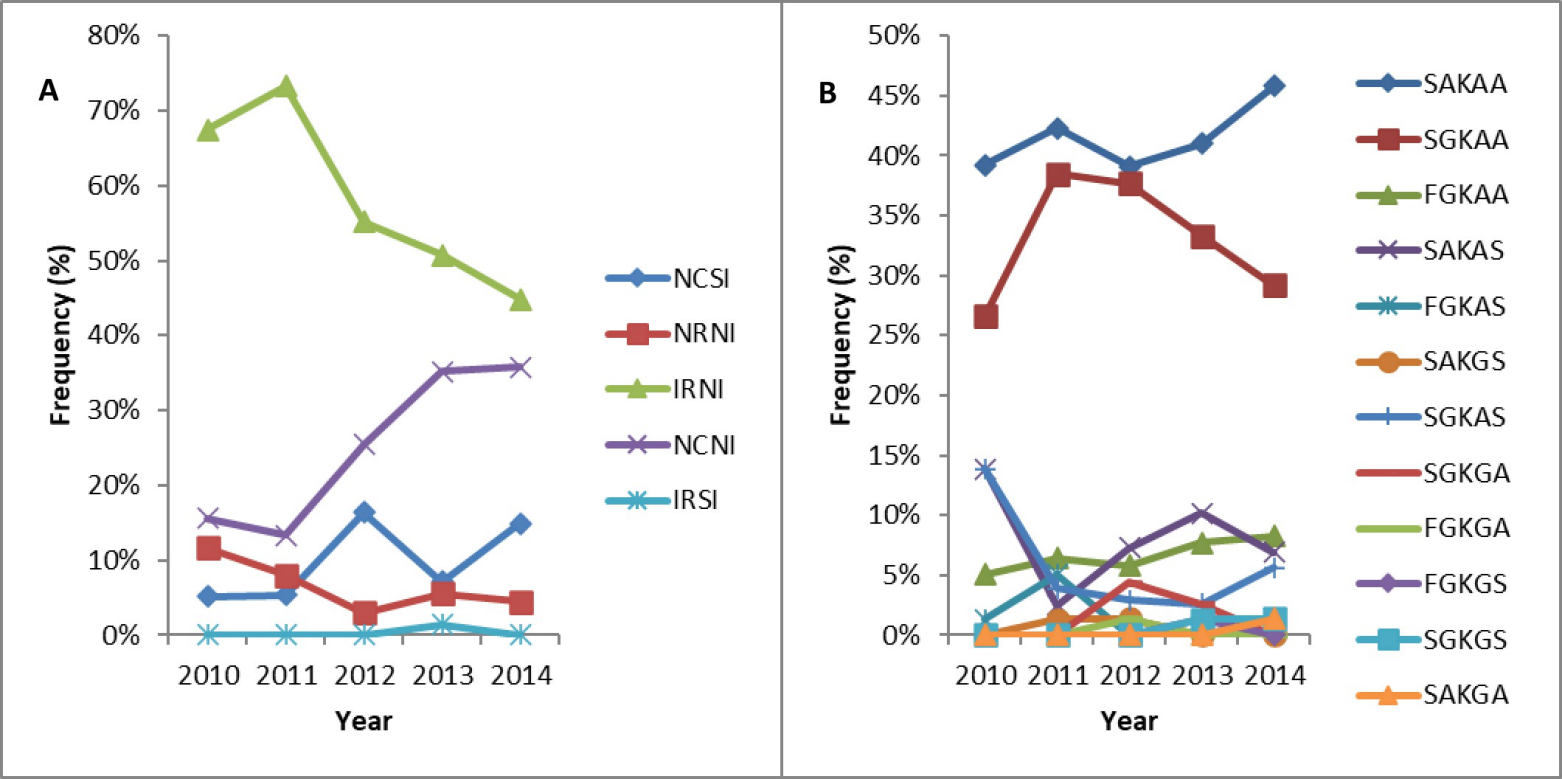
Trends in the frequency of *pfdhfr* and *pdhps* haplotypes at baseline, during and after intervention. **(A)** *pfdhfr*, **(B)** *pfdhps*. Statistical analysis performed using Cuzick’s test for trends.

Overall, five unique *pfdhfr* haplotypes were found across all time points, with four in 2010, 2011, 2012 and 2014 and five in 2013. The most prevalent *pfdhfr* mutant haplotype observed across all time points was I_51_R_59_N_108_I_164_, occurring at frequencies ranging from 44.8–73.3%. The frequency of the triple mutant I_51_R_59_N_108_ associated with pyrimethamine resistance reduced significantly (z = -2.61, P = 0.009) between baseline and post-intervention surveys, during the peak transmission seasons in BY. Twelve unique *pfdhps* haplotypes were found across time points, with six in 2010, seven in 2011 and eight in 2012, 2013 and 2014. The main mutant haplotype observed across all time points was S_436_G_437_K_540_A_581_A_613_, with frequency between 26.6–38.5%. The sulphadoxine resistance-conferring double mutant G_437_E_540_ was absent across all time points, while the frequency of G_437_G_581_ also associated with sulphadoxine resistance showed no trend during the study period (z = 1.39, P = 0.165). The temporal trends in *pfdhfr* and *pfdhps* haplotype frequencies across survey years are presented in Fig 4.

### Frequency of combined *pfcrt* and *pfmdr1* haplotypes

The CQ and AQ resistant *pfcrt/pfmdr1* haplotype, C_72_I_74_E_75_T_76_Y_84_Y_184_Y_1246_ was observed only in two isolates (3%) in 2010 (pre-IRS) and one (1.5%) in 2011, while undetected in 2012, 2013 and 2014. Most isolates genotyped had the C_72_M_74_N_75_K_76_N_84_F_184_D_1246_ haplotype, associated with reduced response to lumefantrine (the partner drug in AL). This haplotype had frequencies of 39.4% in 2010, 36.4% in 2011, 48.3% in 2012, 54.1% in 2013 and 53.3% in 2014. There was however no consistent temporal trend in the haplotype frequency with the IRS intervention in BY (z = -1.32, P = 0.186).

### Frequency of combined *pfdhfr* and *pfdhps* haplotypes

The frequency of combined *pfdhfr* and *pfdhps* haplotypes were also compared to determine the effect of transmission reduction on the synergy between the two genes in conferring resistance to SP after the IRS intervention in BY. The frequency of quadruple I_51_R_59_N_108_G_437_ haplotype associated with partial SP resistance decreased overall across time points. It was prevalent at 29.0% in 2010, 36.5% in 2011, 19.7% in 2012, 26.8% in 2013 and 23.8% in 2014 although this decrease was not significant (z = -1.55, P = 0.120). No quintuple I_51_R_59_N_108_G_437_E_540_ or sextuple I_51_R_59_N_108_G_437_E_540_G_581_ haplotypes associated with full and super resistance to SP respectively were found across survey years in the circulating BY asymptomatic *P*. *falciparum*.

### Frequency of multilocus *pfcrt*, *pfmdr1*, *pfdhfr* and *pfdhps* haplotypes

The linkage between CQ and SP resistant alleles within isolates between 2010 and 2014 in BY (pre-IRS and endline: post-IRS) were assessed based on combined *pfcrt*-T76, *pfmdr1*-Y86, *pfdhfr*-I51, R59 and N108 and *pfdhps*-G437, E540 alleles. Only isolates that were successfully genotyped for all seven markers were included. In all, 30 distinct haplotypes were detected across all time points, with 20 haplotypes in 2010, while 2011, 2012, 2013 and 2014 had 18, 17, 16 and 16 distinct haplotypes, respectively. No septuple mutant haplotypes were detected during the study period, whiles only 1 sextuple haplotype was observed. The number of quintuple, quadruple, triple and double-mutant haplotypes detected over time were 4, 7, 9 and 6, respectively. The highest frequency of all sextuple haplotypes in a particular year was 7.6% (5/66) and this was in 2011; quintuple was 21.8% (14/64) in 2010; quadruple, 33.3% (22/66) in 2011. Similarly, the frequency of the triple and double mutant haplotypes were highest in 2012 and 2013 with values of 40.7% (22/54) and 18.6% (11/59), respectively. Of the most abundant triple mutant haplotypes in a particular year [40.7% (22/54)], majority [77.3% (17/22)] were made up of the pyrimethamine resistance-associated haplotype I_51_R_59_N_108_ and so was the most frequent quadruple haplotypes [33.3% (22/66)], which also had [59.1% (13/22)] carrying the partial SP resistance-conferring haplotype, I_51_R_59_N_108_G_437_. The frequency of the haplotype constructs observed during the study period is shown in S2 Table.

## Discussion

In the light of the emergence of artemisinin resistance in West Cambodia and spread in parts of South-East Asia, surveillance of antimalarial drug resistance in sub-Saharan Africa is important for early detection and containment of emerging resistant strains. Although vector control interventions do not directly shape the malaria parasite population, there remains a large and persistent but mostly neglected asymptomatic *P*. *falciparum* reservoir after these interventions reduce transmission. This circulating reservoir is dominated by infection in partially immune older children and adults [40,41]. Again, although drug pressure is the major driving force for the evolution and or spread of drug resistant parasites, and is often greater in symptomatic infections than in asymptomatic cases [42], the asymptomatic reservoir drives transmission after the interventions and therefore plays a role in the evolution and/or spread of antimalarial resistance.

Our study focused on asymptomatic infections that existed in children under five years in an area of seasonal transmission in Ghana where chloroquine was withdrawn and ACTs implemented in 2005. IPTp coverage with SP during the time period ranged between 54.1–70.7% (IPTp1), 38.7–58.3% (IPTp2), 24.6–41.7% (IPTp3) [43], and an IRS intervention was rolled out in 2011 resulting in a reduction in malaria transmission. By utilizing a repeated cross-sectional study design, we analyzed the prevalence and their trends, if any, in the key polymorphisms of the genes that mediate or augment antimalarial resistance (*pfcrt*, *pfmdr1*, *pfdhfr*, *pfdhps*, and *pfk13*). The study was conducted at the end of the rainy seasons in 2010 (pre-IRS) through to 2014 (endline: post-IRS) in BY District, Ghana and thus, the results reflect the trends in the evolution of drug resistance during the peak transmission seasons. In addition, we analyzed the frequencies of *P*. *falciparum* haplotypes and linked genotypes to determine if there were combinations of genotypes or haplotypes that predominated the area after the IRS rollout that could be used to predict the emergence and/or spread of multidrug resistance in this reservoir. Albeit our study design is limited by the lack of samples from the older children and adults who contribute mostly to the reservoir of infection, children under 5 years old largely contribute to drug resistance as a result of the drug pressure exerted on them. As drug resistance in the asymptomatic group is much lower in comparison with the symptomatic groups [42], children under 5 will be the major source of transmissible resistant parasites especially when transmission intensity has reduced as a result of an enhanced intervention such as IRS as seen in the BY district of Ghana.

In general, there were fluctuations in the prevalence of all the alleles tested and this corroborates with previous studies conducted in Sudan and The Gambia that showed that the prevalence of drug-resistant parasites fluctuate in areas with seasonal malaria transmission [44,45]. Some of the fluctuations nonetheless showed trends over the five-year study period.

Our surveys of the asymptomatic *P*. *falciparum* reservoir in BY observed a decline in the prevalence of key mutants *pfcrt*-T76 and *pfmdr1*-Y86 that mediates CQ resistance [30,46,47] and an increase in the wildtype alleles, following the withdrawal of CQ and IRS rollout. These findings are consistent with observations made in children with clinical infections from Ghana, particularly in the same region [48], and in the Brong Ahafo Region in 2012/2013 [49]. Similarly, other African studies observed a resurgence of the CQ-sensitive alleles following the withdrawal of the drug [50–55]. The return of CQ sensitive parasites may be due to a combination of factors. These include, the fitness cost on the resistant strains in the absence of the drug pressure, the selection of wild-type alleles at both *pfcrt* and *pfmdr1* by the ACT partner drug, lumefantrine, as opposed to the 4-aminoquinolines [CQ and amodiaquine (AQ)] [56–59]. In addition, AQ in ASAQ is still maintaining a sustained pressure on the parasite population in BY as well [60] by selecting for mutant *pfcrt* and *pfmdr1* alleles in circulation. Also there have been sporadic anecdotal evidence of continued private sector sale and use of chloroquine after its removal, however our study was not designed to capture that data.

Drug pressure due to LM and MQ have been associated with the selection of *pfcrt*-K76, *pfmdr1*-N86, F184 and D1246 [61–63]. On the other hand, the selection of *pfcrt*-T76, *pfmdr1*-Y86, Y184 and Y1246 have been attributed to decreased AQ sensitivity [64–66]. A trend in lower prevalence of *pfcrt*-T76 and *pfmdr1*-Y_86_Y_184_Y_1246_ as compared to *pfcrt*-K76 and *pfmdr1*- N_86_F_184_D_1246_ were observed in BY with the enhanced IRS rollout over the study period. This is consistent with prevalence (35–53% and 4–6%, respectively) recorded in clinical settings in Ghana, between 2003 to 2010 [48]. These findings suggest that LM resistant parasites are evolving faster than AQ resistance parasites in Ghana. This is not surprising because, since its introduction in 2007 as an additional first-line treatment for uncomplicated malaria for patients who adversely reacted to ASAQ, [67], AL has become the preferred ACT of the two [68,69].

Contrary to previous studies in Ghana (with no IRS intervention) where the *pfmdr1*-N_86_F_184_D_1246_ associated with LM resistance maintained a high prevalence over time [70] or saw a trend in increasing prevalence [48], we observed a trend in the reduction of *pfmdr1*-N_86_F_184_D_1246_ and *pfmdr1-*Y_86_Y_184_Y_1246_ frequencies, although most parasites isolates carried *pfmdr1*-N_86_F_184_D_1246_ than the *pfmdr1-*Y_86_Y_184_Y_1246._ Our findings indicate that with the IRS intervention that culminated in a reduction in malaria transmission in BY, there was a delay in the selection of mutant alleles associated with LM and AQ treatment failure in the presence of the drug pressures. This could be explained by reductions in: the population size of the mosquito vectors, circulating mutant parasites [17], the number of parasite clones per host and the level of drug use in the population [17,23] as transmission intensity reduced in BY district.

Multidrug resistance has predominated South East Asia where numerous strategies for *P*. *falciparum* elimination have been implemented [71–77]. This is because inbreeding in the clonal parasite populations has prevailed and drug resistant gene combinations in the population have become stable and rapidly increase in frequency [20,25]. We observed only one sextuple mutant parasite and no septuple, an indication that there was very little linkage between mutant alleles associated with the ACT partner drugs and anti-folate drugs currently deployed, and no immediate threat of multidrug resistance in BY nine years after ACTs were deployed as first line treatment of uncomplicated malaria and the enhanced IRS rollout in 2014.

The observed higher prevalence of *pfdhfr* mutations as compared to the *pfdhps* in this study is not surprising, as the mechanism of SP resistance in *P*. *falciparum* is a continuous stepwise process occurring first in *pfdhfr* gene, after which *pfdhps* mutation is next selected for, but only when most parasite population carry at least a double but usually triple *pfdhfr* mutant alleles [34,78,79]. We observed a high prevalence of S108N (83.8% - 95.0%), C59R (49.3% - 81.3%) and N51I (44.8% - 73.3%) followed by the A437G (46.2% - 54.2%) mutations, and eventually A613S, S436F and A581G were implicated. These findings are consistent with results reported in other settings in Africa [53,80–83]. Similar to the report by Mockenhaupt and colleagues, we detected *pfdhps-*G581 and S613 among parasites from the same region in Ghana (Northern Region) [84]. Like studies conducted in other regions of Ghana, Kenya and Senegal [53,85,86], we have reported the F436 in the BY district after the intense IRS rollout. This thus emphasizes the need for continuous monitoring of these previously rare markers for timely response, as they have been shown to confer high level resistance to SP treatment [87].

This study also found *pfdhfr*- I_51_R_59_N_108_I_164_ (44.8–73.3%) and *pfdhps*-S_436_G_437_K_540_A_581_A_613_ (26.6–38.5%) as the most prevalent SP resistant haplotypes across all time points, and this corroborates with finding from Cameroon and Gabon [88,89]. Ultimately, the absence of SP super resistant parasites bearing the quintuple mutants I_51_R_59_N_108_G_437_E_540_ haplotype as a consequence of the absence of *pfdhps-*E540 gives strong justification for the continued use of SP for IPTp in Ghana.

Furthermore, the critical *pfk13* gene mutation C580Y, which is associated with delayed clearance of ART in Southeast Asia [35,90–92], was not detected across any of the time points. Similarly, the other 8 validated Southeast Asian K13 alleles associated with ART resistance (F446I, N458Y/I, M476I/V, Y493H, R539T, I543T, P553L, R561H), are also rare in Africa [93,94]. The African K13 mutant alleles (including G449A/D, V520A, S522C, C542Y, G544R, G545E, A557S, R561C, A578S, A617V/T, V637A/I/D, G638R) either do not show evidence of selection in the parasite population or have not yet been associated with clinical artemisinin resistance [37,90,94,95]. This thus corroborates with findings that resistance to ART has not been established in Africa [95–97]. Continuous surveillance on the continent is however essential for early detection of resistance to ART.

A limitation to the current study is that, our findings are from one geographical site with a common transmission setting. However, to truly appreciate how transmission intensity changes influence drug resistance, it would be prudent to have obtained and compared data from different geographical areas of varying transmission intensities (preferably from low, moderate and high transmission areas) to that of data from intervention studies like the present study and to have allowed more time to elapse.

## Conclusion

In summary, the validated K13 resistance mutation Y580, was not detected in the study district. The low prevalence of SP super-resistant parasites bearing the quintuple mutants I_51_R_59_N_108_G_437_E_540_ haplotype supports the continued use of SP for IPTp in Ghana. This study demonstrated that the use of effective drug treatment policy, coupled with reduction in transmission intensity as a result of vector control (IRS scale-up) led to the reduction in the prevalence of drug resistant markers associated with ACT partner drugs: AQ and LM. Further empirical evidence from different geographical settings of varying transmission intensities and in older children and adults is needed to enhance the understanding of the effect of transmission reduction on the evolution of drug resistance in asymptomatic populations.

## Supporting information

S1 Table Alleles proportions across survey years. (DOCX)

S2 Table Multilocus *pfcrt*, *pfmdr1*, *pfdhfr and pfdhps* haplotypes across survey years. (DOCX)

S1 Data (XLS)

## References

[1] WHO. World Malaria Report. Geneva: World Health Organization; 2019.

[2] Froeschl G, Saathoff E, Kroidl I, Berens-Riha N, Clowes P, Maboko L, et al Reduction of malaria prevalence after introduction of artemisinin-combination-therapy in Mbeya Region, Tanzania: Results from a cohort study with 6773 participants. Malar J. 2018;17 10.1186/s12936-018-2389-z 29940968 PMC6019526

[3] Hetzel MW, Reimer LJ, Gideon G, Koimbu G, Barnadas C, Makita L, et al Changes in malaria burden and transmission in sentinel sites after the roll-out of long-lasting insecticidal nets in Papua New Guinea. Parasites and Vectors. 2016;9 10.1186/s13071-016-1635-x 27301964 PMC4908799

[4] Katureebe A, Zinszer K, Arinaitwe E, Rek J, Kakande E, Charland K, et al Measures of Malaria Burden after Long-Lasting Insecticidal Net Distribution and Indoor Residual Spraying at Three Sites in Uganda: A Prospective Observational Study. PLoS Med. 2016;13 10.1371/journal.pmed.1002167 27824885 PMC5100985

[5] Pluess B, Tanser FC, Lengeler C, Sharp BL. Indoor residual spraying for preventing malaria (Review). Cochrane database Syst Rev. 2010; 10.1002/14651858.CD006657.pub2 PMC6532743 20393950

[6] Afari EA, Appawu M, Dunyo S, Baffoe-Wilmot A, Nkrumah FK. Malaria infection, morbidity and transmission in two ecological zones Southern Ghana. Afr J Health Sci. 1995;2: 312–315. 12160443

[7] Ghana Health Service. National Malaria Control Programme: 2015 Annual Report. Ghana Heal Serv. 2016; 1–70. Available: https://www.ghanahealthservice.org/downloads/NMCP_2015_Annual_Report.

[8] Ministry of Health G. Guidelines for Case Management of Malaria in Ghana. Minist Heal. 2014; 1–76. Available: https://www.ghanahealthservice.org/downloads/guidelineforcasemanagement.

[9] Ghana Statistical Service G, Ghana Health Service G, ICF I. Ghana Demographic and Health Survey 2014. GSS, GHS ICF Int Rockville, Maryland, USA; 2015;

[10] Ghana Statistical Sercive G, Ghana Health Service G, ICF. Ghana Malaria Indicator Survey 2016. GSS, GHS ICF Accra, Ghana and Rockville, Maryland, USA; 2017;

[11] National Malaria Control Programme N, University of Health & Allied Sciences U, AGA Malaria Control Programme A, World Health Organization W, Inform Project K. An Epidemiological Profile of Malaria and its Control in Ghana. MOH, RBM DID. 2013;

[12] Abuaku B, Ahorlu C, Psychas P, Ricks P, Oppong S, Mensah S, et al Impact of indoor residual spraying on malaria parasitaemia in the Bunkpurugu-Yunyoo District in northern Ghana. Parasites and Vectors. 2018;11 10.1186/s13071-018-3130-z 30352613 PMC6199755

[13] Chen I, Clarke SE, Gosling R, Hamainza B, Killeen G, Magil A, et al “Asymptomatic” Malaria: A Chronic and Debilitating Infection That Should Be Treated. PLoS Med. 2016;13 10.1371/journal.pmed.1001942 26783752 PMC4718522

[14] Lin JT, Saunders DL, Meshnick SR. The role of submicroscopic parasitemia in malaria transmission: what is the evidence? Trends Parasitol. 2014;30: 183–190. 10.1016/j.pt.2014.02.004 24642035 PMC4049069

[15] Bousema T, Okell L, Felger I, Drakeley C. Asymptomatic malaria infections: detectability, transmissibility and public health relevance. Nat Rev Microbiol. 2014;12: 833–840. 10.1038/nrmicro3364 25329408

[16] Talisuna AO, Langi P, Bakyaita N, Egwang T, Mutabingwa TK, Watkins W, et al Intensity of malaria transmission, antimalarial-drug use and resistance in Uganda: what is the relationship between these three factors? Trans R Soc Trop Med Hyg. 2002;96: 310–317. 10.1016/s0035-9203(02)90108-2 12174786

[17] Alifrangis M, Lemnge MM, Segeja MD, Magesa SM, Khalil IF, Bygbjerg IBC. Increasing prevalence of wildtypes in the dihydrofolate reductase gene of plasmodium falciparum in an area with high levels of sulfadoxine / pyrimethamine resistance after introduction of treated bed nets. Am J Trop Med Hyg. 2003;69: 238–243. 10.4269/ajtmh.2003.69.238 14628937

[18] Mharakurwa S, Mutambu SL, Mudyiradima R, Chimbadzwa T, Chandiwana SK, Day KP. Association of house spraying with suppressed levels of drug resistance in Zimbabwe. Malar J. 2004;3 10.1186/1475-2875-3-35 15491495 PMC535889

[19] Ariey F, Robert V. The puzzling links between malaria transmission and drug resistance. Trends Parasitol. 2003;19: 158–160. 10.1016/s1471-4922(03)00054-0 12689643

[20] Schmidt KF. Inbred Parasites May Spur Resistance. Science (80-). 1995;269: 1670 10.1126/science.7569890 7569890

[21] Diallo DA, Sutherland C, Nebié I, Konaté AT, Ord R, Pota H. Sustained use of insecticide-treated curtains is not associated with greater circulation of drug-resistant malaria parasites, or with higher risk of treatment failure among children with uncomplicated malaria in Burkina Faso. Am J Trop Med Hyg. 2007;76: 237–244. 10.4269/ajtmh.2007.76.237 17297030

[22] Shah M, Kariuki S, Vanden Eng J, Blackstock AJ, Garner K, Gimnig JE, et al Effect of Transmission Reduction by Insecticide-Treated Bednets (ITNs) on Antimalarial Drug Resistance in Western Kenya. PLoS One. 2011;6 10.1371/journal.pone.0026746 22096496 PMC3214025

[23] Shah M, Omosun Y, Lal A, Odero C, Gatei W, Otieno K, et al Assessment of molecular markers for anti-malarial drug resistance after the introduction and scale-up of malaria control interventions in western Kenya. Malar J. 2015;14 10.1186/s12936-015-0588-4 25889220 PMC4331436

[24] Talisuna AO, Langi P, Mutabingwa TK, Van Marck E, Speybroeck N, Egwang TG, et al Intensity of transmission and spread of gene mutations linked to chloroquine and sulphadoxine-pyrimethamine resistance in falciparum malaria. Int J Parasitol. 2003;33: 1051–1058. 10.1016/s0020-7519(03)00156-5 13129527

[25] Hastings IM. Malaria control and the evolution of drug resistance: An intriguing link. Trends Parasitol. 2003;19: 70–73. 10.1016/s1471-4922(02)00017-x 12586474

[26] Sidhu A, Verdier-Pinard D, Fidock DA. Chloroquine resistance in Plasmodium falciparum malaria parasites conferred by pfcrt mutations. Science (80-). 2002;298: 210–213. 10.1126/science.1074045 12364805 PMC2954758

[27] Warhurst DC. A Molecular Marker for Chloroquine-Resistant Falciparum Malaria. N Engl J Med. 2001;344: 299–302. 10.1056/NEJM200101253440411 11172160

[28] Duraisingh MT, Jones P, Sambou I, Von Seidlein L, Pinder M, Warhurst DC. The tyrosine-86 allele of the pfmdr1 gene of Plasmodium falciparum is associated with increased sensitivity to the anti-malarials mefloquine and artemisinin. Mol Biochem Parasitol. 2000;108: 13–23. 10.1016/s0166-6851(00)00201-2 10802315

[29] Mwai L, Kiara SM, Abdirahman A, Pole L, Rippert A, Diriye A, et al In vitro activities of piperaquine, lumefantrine, and dihydroartemisinin in Kenyan plasmodium falciparum isolates and polymorphisms in pfcrt and pfmdr1. Antimicrob Agents Chemother. 2009;53: 5069–5073. 10.1128/AAC.00638-09 19770282 PMC2786317

[30] Reed MB, Saliba KJ, Caruana SR, Kirk K, Cowman AF. Pgh1 modulates sensitivity and resistance to multiple antimalarials in Plasmodium falciparum. Nature. 2000;403: 906–909. 10.1038/35002615 10706290

[31] Hyde JE. Mechanisms of resistance of Plasmodium falciparum to antimalarial drugs. Elsevier. 2002;4: 165–174. 10.1016/s1286-4579(01)01524-6 11880048

[32] Gregson A, Plowe C V. Mechanisms of Resistance of Malaria Parasites to Antifolates. Pharmacol Rev. 2005;57: 117–145. 10.1124/pr.57.1.4.117 15734729

[33] Hankins EG, Warhurst DC, Sibley CH. Novel alleles of the Plasmodium falciparum dhfr highly resistant to pyrimethamine and chlorcycloguanil, but not WR99210. Mol Biochem Parasitol. 2001;117: 91–102. 10.1016/s0166-6851(01)00335-8 11551635

[34] Sibley CH, Hyde JE, Sims PF, Plowe C V, Kublin JG, Mberu EK, et al Pyrimethamine-sulfadoxine resistance in Plasmodium falciparum: what next? Trends Parasitol. 2001;17: 582–588. 10.1016/s1471-4922(01)02085-2 11756042

[35] Ariey F, Witkowski B, Amaratunga C, Beghain J, Ma L, Lim P, et al A molecular marker of artemisinin-resistant Plasmodium falciparum malaria. Nature. 2014;505: 50–55. 10.1038/nature12876 24352242 PMC5007947

[36] Straimer J, Gnädig NF, Witkowski B, Khim N, Zhang L, Lam S, et al K13-propeller mutations confer artemisinin resistance in Plasmodium falciparum clinical isolates. Scienc. 2015;347: 428–431. 10.1126/science.1260867 25502314 PMC4349400

[37] Ménard D, Khim N, Beghain J, Adegnika AA, Shafiul‑Alam M, Amodu O, et al A Worldwide Map of Plasmodium falciparum K13-Propeller Polymorphisms. N Engl J Med. 2016;374: 2453–2464. 10.1056/NEJMoa1513137 27332904 PMC4955562

[38] Daniels R, Ndiaye D, Wall M, McKinney J, Séne PD, Sabeti PC, et al Rapid, field-deployable method for genotyping and discovery of single-nucleotide polymorphisms associated with drug resistance in Plasmodium falciparum. Antimicrob Agents Chemother. 2012;56: 2976–2986. 10.1128/AAC.05737-11 22430961 PMC3370755

[39] Mukherjee A, Bopp S, Magistrado P, Wong W, Daniels R, Demas A, et al Artemisinin resistance without pfkelch13 mutations in Plasmodium falciparum isolates from Cambodia. Malar J. 2017;16 10.1186/s12936-017-1845-5 28494763 PMC5427620

[40] Gonçalves BP, Kapulu MC, Sawa P, Guelbéogo WM, Tiono AB, Grignard L, et al Examining the human infectious reservoir for Plasmodium falciparum malaria in areas of differing transmission intensity. Nat Commun. 2017;8 10.1038/s41467-017-01270-4 29074880 PMC5658399

[41] Coalson JE, Cohee LM, Buchwald AG, Nyambalo A, Kubale J, Seydel KB, et al Simulation models predict that school ‑ age children are responsible for most human ‑ to ‑ mosquito Plasmodium falciparum transmission in southern Malawi. Malar J. 2018;17 10.1186/s12936-018-2295-4 29615044 PMC5883608

[42] Hastings IM, D’Alessandro U. Modelling a predictable disaster: The rise and spread of drug-resistant malaria. Parasitol Today. 2000;16: 340–347. 10.1016/s0169-4758(00)01707-5 10900482

[43] Ghana Health Service G. National Malaria Control Programme: 2014 Annual Report. GHS. 2015;

[44] Babiker HA, Satti G, Walliker D. Genetic changes in the population of Plasmodium falciparum in a Sudanese village over a three-year period. Am J Trop Med Hyg. 1995;53: 7–15. 10.4269/ajtmh.1995.53.7 7625537

[45] Ord R, Alexander N, Dunyo S, Hallett R, Jawara M, Targett G, et al Seasonal Carriage of pfcrt and pfmdr1 Alleles in Gambian Plasmodium falciparum Imply Reduced Fitness of Chloroquine-Resistant Parasites. J Infect Dis. 2007;196: 1613–1619. 10.1086/522154 18008244

[46] Fidock DA, Nomura T, Talley AK, Cooper RA, Dzekunov SM, Ferdig MT, et al Mutations in the *P*. *falciparum* digestive vacuole transmembrane protein PfCRT and evidence for their role in chloroquine resistance. Mol Cell. 2000;6: 861–871. 10.1016/s1097-2765(05)00077-8 11090624 PMC2944663

[47] Babiker HA, Pringle SJ, Abdel‐Muhsin A, Mackinnon M, Hunt P, Walliker D. High‐Level Chloroquine Resistance in Sudanese Isolates of Plasmodium falciparum Is Associated with Mutations in the Chloroquine Resistance Transporter Gene pfcrt and the Multidrug Resistance Gene pfmdr1. J Infect Dis. 2001;183: 1535–1538. 10.1086/320195 11319692

[48] Duah NO, Matrevi SA, de Souza DK, Binnah DD, Tamakloe MM, Opoku VS, et al Increased pfmdr1 gene copy number and the decline in pfcrt and pfmdr1 resistance alleles in Ghanaian Plasmodium falciparum isolates after the change of anti-malarial drug treatment policy. Malar J. Malaria Journal; 2013;12 10.1186/1475-2875-12-377 24172030 PMC3819684

[49] Abugri J, Ansah F, Asante KP, Opoku CN, Amenga-Etego LA, Awandare GA. Prevalence of chloroquine and antifolate drug resistance alleles in Plasmodium falciparum clinical isolates from three areas in Ghana. AAS Open Res. 2018;1 10.12688/aasopenres.12825.2 32382694 PMC7185243

[50] Kublin JG, Cortese JF, Njunju M, Mukadam RAG, Wirima JJ, Kazembe PN, et al Reemergence of Chloroquine-Sensitive Plasmodium falciparum Malaria after Cessation of Chloroquine Use in Malawi. J Infect Dis. 2003;187: 1870–1875. 10.1086/375419 12792863

[51] Raman J, Mauff K, Muianga P, Mussa A, Maharaj R, Barnes KI. Five Years of Antimalarial Resistance Marker Surveillance in Gaza Province, Mozambique, Following Artemisinin- Based Combination Therapy Roll Out. PLoS One. 2011;6 10.1371/journal.pone.0025992 22022487 PMC3195082

[52] Mwai L, Ochong E, Abdirahman A, Kiara SM, Ward S, Kokwaro G, et al Chloroquine resistance before and after its withdrawal in Kenya. Malar J. 2009;10 10.1186/1475-2875-8-106 PMC2694831 19450282

[53] Wurtz N, Fall B, Pascual A, Diawara S, Sow K, Baret E, et al Prevalence of molecular markers of Plasmodium falciparum drug resistance in Dakar, Senegal. Malar J. 2012;11 10.1186/1475-2875-11-197 22694921 PMC3470961

[54] Moyeh MN, Njimoh DL, Evehe MS, Ali IM, Nji AM, Nkafu DN, et al Effects of Drug Policy Changes on Evolution of Molecular Markers of Plasmodium falciparum Resistance to Chloroquine, Amodiaquine, and Sulphadoxine-Pyrimethamine in the South West Region of Cameroon. Malar Res Treat. 2018;10 10.1155/2018/7071383 PMC5954917 29854394

[55] Eyase FL, Akala HM, Ingasia L, Cheruiyot A, Omondi A, Okudo C, et al The Role of Pfmdr1 and Pfcrt in Changing Chloroquine, Amodiaquine, Mefloquine and Lumefantrine Susceptibility in Western-Kenya P. falciparum Samples during 2008–2011. PLoS One. 2013;8 10.1371/journal.pone.0064299 PMC3652850 23675533

[56] Veiga MI, Dhingra SK, Henrich PP, Straimer J, Gnädig N, Uhlemann A-C, et al Globally prevalent PfMDR1 mutations modulate Plasmodium falciparum susceptibility to artemisinin-based combination therapies. Nat Commun. 2016;7: 11553 10.1038/ncomms11553 27189525 PMC4873939

[57] Humphreys GS, Merinopoulos I, Ahmed J, Whitty CJM, Mutabingwa TK, Sutherland CJ, et al Amodiaquine and Artemether-Lumefantrine Select Distinct Alleles of the Plasmodium falciparum mdr1 Gene in Tanzanian Children Treated for Uncomplicated Malaria. Antimicrob Agents Chemother. 2007;51: 991–997. 10.1128/AAC.00875-06 17194834 PMC1803116

[58] Somé AF, Séré YY, Dokomajilar C, Zongo I, Rouamba N, Greenhouse B, et al Selection of known plasmodium falciparum resistance-mediating polymorphisms by artemether-lumefantrine and amodiaquine-sulfadoxine-pyrimethamine but not dihydroartemisinin-piperaquine in Burkina Faso. Antimicrob Agents Chemother. 2010;54: 1949–1954. 10.1128/AAC.01413-09 20231394 PMC2863637

[59] Rosenthal PJ. The interplay between drug resistance and fitness in malaria parasites. Mol Microbiol. 2013;89: 1025–1038. 10.1111/mmi.12349 23899091 PMC3792794

[60] Ochong E, Broek I van den, Keus K. Short Report: Association between chloroquine and amodiaquine resistance and allelic variation in the Plasmodium falciparum multiple drug resistance 1 gene and the chloroquine resistance transporter gene in isolates from the upper Nile in southern Sudan. Am J Trop Med Hyg. 2003;69: 184–187. 13677373

[61] Conrad MD, Leclair N, Arinaitwe E, Wanzira H, Kakuru A, Bigira V, et al Comparative Impacts Over 5 Years of Artemisinin-Based Combination Therapies on Plasmodium falciparum Polymorphisms That Modulate Drug Sensitivity in Ugandan Children. J Infect Dis. 2014;210: 344–353. 10.1093/infdis/jiu141 24610872 PMC4110461

[62] Malmberg M, Ferreira PE, Tarning J, Ursing J, Ngasala B, Björkman A, et al Plasmodium falciparum Drug Resistance Phenotype as Assessed by Patient Antimalarial Drug Levels and Its Association With pfmdr1 Polymorphisms. J Infect Dis. 2013;207: 842–847. 10.1093/infdis/jis747 23225895 PMC3563306

[63] Baraka V, Mavoko HM, Nabasumba C, Francis F, Lutumba P, Alifrangis M, et al Impact of treatment and re-treatment with amodiaquine on selection of Plasmodium falciparum multidrug resistance gene-1 polymorphisms in the Democratic Republic of Congo and Uganda. PLoS One. 2018;13 10.1371/journal.pone.0191922 29390014 PMC5794077

[64] Tinto H, Guekoun L, Zongo I, Guiguemde RT, D’Alessandro U, Ouedraogo JB. Chloroquine-resistance molecular markers (Pfcrt T76 and Pfmdr-1 Y86) and amodiaquine resistance in Burkina Faso. Trop Med Int Health. 2008;13: 238–240. 10.1111/j.1365-3156.2007.01995.x 18304270

[65] Danquah I, Coulibaly B, Meissner P, Petruschke I, Müller O, Mockenhaupt FP. Selection of pfmdr1 and pfcrt alleles in amodiaquine treatment failure in north-western Burkina Faso. Acta Trop. 2010;114: 63–66. 10.1016/j.actatropica.2009.12.008 20060374

[66] Nsobya SL, Kiggundu M, Nanyunja S, Joloba M, Greenhouse B, Rosenthal PJ. In Vitro Sensitivities of Plasmodium falciparum to Different Antimalarial Drugs in Uganda. Antimicrob Agents Chemother. 2010;54: 1200–1206. 10.1128/AAC.01412-09 20065051 PMC2825959

[67] Ministry of Health. Guidelines for case management of malaria in Ghana. MOH 2014;

[68] Afriyie DK, Amponsah SK, Antwi R, Nyoagbe SY. Prescribing trend of antimalarial drugs at the Ghana Police Hospital. J Infect Dev Ctries. 2015;9: 409–415. 10.3855/jidc.5578 25881531

[69] Abuaku B, Quashie NOD, Quaye L, Matrevi SA, Quashie N, Gyasi A, et al Therapeutic efficacy of artesunate–amodiaquine and artemether–lumefantrine combinations for uncomplicated malaria in 10 sentinel sites across Ghana: 2015–2017. Malar J. 2019;18 10.1186/s12936-019-2848-1 31234874 PMC6591907

[70] Mensah BA, Aydemir O, Myers-Hansen JL, Opoku M, Hathaway J, Marsh PW, et al Antimalarial drug resistance profiling of Plasmodium falciparum infections in Ghana using molecular inversion probes and next generation sequencing. Antimicrob Agents Chemother. 2020; 10.1128/AAC.01423-19 31932374 PMC7179265

[71] Mackinnon MJ, Hastings IM. The evolution of multiple drug resistance in malaria parasites. Trans R Soc Trop Med Hyg. 1998;92: 188–195. 10.1016/s0035-9203(98)90745-3 9764331

[72] Hastings IM, Mackinnon MJ. The emergence of drug-resistant malaria. Parasitology. 1998;117: 411–417. 10.1017/s0031182098003291 9836305

[73] Wongsrichanalai C, Pickard AL, Wernsdorfer WH, Meshnick SR. Epidemiology of drug-resistant malaria. Lancet Infect Dis. 2002;2: 209–218. 10.1016/s1473-3099(02)00239-6 11937421

[74] Wernsdorfer WH. Epidemiology of drug resistance in malaria. Acta Trop. 1994;56: 143–156. 10.1016/0001-706x(94)90060-4 8203301

[75] Amato R, Lim P, Miotto O, Amaratunga C, Dek D, Pearson RD, et al Genetic markers associated with dihydroartemisinin–piperaquine failure in Plasmodium falciparum malaria in Cambodia: a genotype–phenotype association study. Lancet Infect Dis. 2017;17: 164–173. 10.1016/S1473-3099(16)30409-1 27818095 PMC5564489

[76] Amato R, Pearson RD, Almagro-Garcia J, Amaratunga C, Lim P, Suon S, et al Origins of the current outbreak of multidrug-resistant malaria in southeast Asia: A retrospective genetic study. Lancet Infect Dis. 2018;18: 337–345. 10.1016/S1473-3099(18)30068-9 29398391 PMC5835763

[77] Miotto O, Almagro-Garcia J, Manske M, Macinnis B, Campino S, Rockett K, et al Multiple populations of artemisinin-resistant Plasmodium falciparum in Cambodia. Nat Genet. 2013;45: 648–655. 10.1038/ng.2624 23624527 PMC3807790

[78] Nzila AM, Mberu EK, Sulo J, Dayo H, Winstanley PA, Sibley CH, et al Towards an Understanding of the Mechanism of Pyrimethamine-Sulfadoxine Resistance in Plasmodium falciparum: Genotyping of Dihydrofolate Reductase and Dihydropteroate Synthase of Kenyan Parasites. Antimicrob Agents Chemother. 2000;44: 991–996. 10.1128/aac.44.4.991-996.2000 10722502 PMC89803

[79] Nzila AM, Nduati E, Mberu EK, Sibley CH, Monks SA, Winstanley PA, et al Molecular Evidence of Greater Selective Pressure for Drug Resistance Exerted by the Long-Acting Antifolate Pyrimethamine / Sulfadoxine Compared with the Shorter-Acting Chlorproguanil / Dapsone on Kenyan Plasmodium falciparum. J Infect Dis. 2000;181: 2023–2028. 10.1086/315520 10837185

[80] Jiang T, Chen J, Fu H, Wu K, Yao Y, Urbano J, et al High prevalence of Pfdhfr–Pfdhps quadruple mutations associated with sulfadoxine–pyrimethamine resistance in Plasmodium falciparum isolates from Bioko Island, Equatorial Guinea. Malar J. 2019;18 10.1186/s12936-019-2734-x 30914041 PMC6434785

[81] Ndiaye D, Daily JP, Sarr O, Ndir O, Gaye O, Mboup S, et al Mutations in Plasmodium falciparum dihydrofolate reductase and dihydropteroate synthase genes in Senegal. Trop Med Int Heal. 2005;10: 1176–1179. 10.1111/j.1365-3156.2005.01506.x 16262743 PMC2582373

[82] Ndong Ngomo JM, Mawili-Mboumba DP, M’Bondoukwe NP, Nikiéma Ndong Ella R, Bouyou Akotet MK. Increased Prevalence of Mutant Allele Pfdhps 437G and Pfdhfr Triple Mutation in Plasmodium falciparum Isolates from a Rural Area of Gabon, Three Years after the Change of Malaria Treatment Policy. Malar Res Treat. 2016;2016 10.1155/2016/9694372 27190671 PMC4852121

[83] Siame MN, Mharakurwa S, Chipeta J, Thuma P, Michelo C. High prevalence of dhfr and dhps molecular markers in Plasmodium falciparum in pregnant women of Nchelenge district, Northern Zambia. Malar J. 2015;14 10.1186/s12936-015-0676-5 25943379 PMC4425916

[84] Mockenhaupt FP, Bousema JT, Eggelte TA, Schreiber J, Ehrhardt S. Plasmodium falciparum dhfr but not dhps mutations associated with sulphadoxine-pyrimethamine treatment failure and gametocyte carriage in northern Ghana. Trop Med Int Health. 2005;10: 901–908. 10.1111/j.1365-3156.2005.01471.x 16135198

[85] Alam T, Souza DK De, Vinayak S, Griffing SM, Poe AC, Duah NO, et al Selective Sweeps and Genetic Lineages of Plasmodium falciparum Drug -Resistant Alleles in Ghana. J Infect Dis. 2011;203: 220–227. 10.1093/infdis/jiq038 21288822 PMC3071065

[86] Oguike MC, Falade CO, Shu E, Enato IG, Watila I, Baba ES, et al Molecular determinants of sulfadoxine-pyrimethamine resistance in Plasmodium falciparum in Nigeria and the regional emergence of dhps 431V. Int J Parasitol Drugs Drug Resist. 2016;6: 220–229. 10.1016/j.ijpddr.2016.08.004 27821281 PMC5094156

[87] Bacon DJ, Tang D, Salas C, Roncal N, Lucas C, Gerena L, et al Effects of point mutations in Plasmodium falciparum dihydrofolate reductase and dihydropterate synthase genes on clinical outcomes and in vitro susceptibility to sulfadoxine and pyrimethamine. PLoS One. 2009;4 10.1371/journal.pone.0006762 19707564 PMC2728505

[88] Apinjoh TO, Mugri RN, Miotto O, Chi HF, Tata RB, Anchang-kimbi JK, et al Molecular markers for artemisinin and partner drug resistance in natural Plasmodium falciparum populations following increased insecticide treated net coverage along the slope of mount Cameroon: cross-sectional study. Infect Dis Poverty. 2017;6 10.1186/s40249-017-0350-y 29110722 PMC5674235

[89] Voumbo-Matoumona DF, Kouna LC, Madamet M, Maghendji-Nzondo S, Pradines B, Lekana-Douki JB. Prevalence of Plasmodium falciparum antimalarial drug resistance genes in Southeastern Gabon from 2011 to 2014. Infect Drug Resist. 2018;11: 1329–1338. 10.2147/IDR.S160164 30214253 PMC6118251

[90] Kamau E, Campino S, Amenga-Etego L, Drury E, Ishengoma D, Johnson K, et al K13-propeller polymorphisms in plasmodium falciparum parasites from sub-saharan Africa. J Infect Dis. 2015;211: 1352–1355. 10.1093/infdis/jiu608 25367300 PMC4827505

[91] Miotto O, Amato R, Ashley EA, Macinnis B, Almagro-garcia J, Amaratunga C, et al Genetic architecture of artemisinin-resistant Plasmodium falciparum. Nat Genet. 2015;47: 226–234. 10.1038/ng.3189 25599401 PMC4545236

[92] Takala-Harrison S, Jacob CG, Arze C, Cummings MP, Silva JC, Khanthavong M, et al Independent Emergence of Artemisinin Resistance Mutations Among Plasmodium falciparum in Southeast Asia. J Infect Dis. 2015;211: 670–679. 10.1093/infdis/jiu491 25180241 PMC4334802

[93] Chhibber-Goel J, Sharma A. Profiles of Kelch mutations in Plasmodium falciparum across South Asia and their implications for tracking drug resistance. Int J Parasitol Drugs Drug Resist. Elsevier; 2019;11: 49–58. 10.1016/j.ijpddr.2019.10.001 31606696 PMC6796718

[94] Matrevi SA, Opoku-Agyeman P, Quashie NB, Bruku S, Abuaku B, Koram KA, et al Plasmodium falciparum Kelch Propeller Polymorphisms in Clinical Isolates from Ghana from 2007 to 2016. Antimicrob Agents Chemother. 2019;63 10.1128/AAC.00802-19 31427297 PMC6811449

[95] Taylor SM, Parobek CM, De Conti DK, Kayentao K, Coulibaly SO, Greenwood BM, et al Absence of putative artemisinin resistance mutations among Plasmodium falciparum in sub-Saharan Africa: A molecular epidemiologic study. J Infect Dis. 2015;211: 680–688. 10.1093/infdis/jiu467 25180240 PMC4402372

[96] Boussaroque A, Fall B, Madamet M, Camara C, Benoit N, Fall M, et al Emergence of mutations in the K13 propeller gene of Plasmodium falciparum isolates from Dakar, Senegal, in 2013–2014. Antimicrob Agents Chemother. 2016;60: 624–627. 10.1128/AAC.01346-15 26503652 PMC4704212

[97] Li J, Chen J, Xie D, Eyi UM, Matesa RA, Ondo Obono MM, et al Limited artemisinin resistance-associated polymorphisms in Plasmodium falciparum K13-propeller and PfATPase6 gene isolated from Bioko Island, Equatorial Guinea. Int J Parasitol Drugs Drug Resist. 2016;6: 54–59. 10.1016/j.ijpddr.2015.11.002 27054064 PMC4805774

