# Supplementary material for: Assessment of antimalarial drug resistant markers in asymptomatic Plasmodium falciparum infections after 4 years of indoor residual spraying in Northern Ghana
Source: PLoS One. 2020 Dec 7;15(12):e0233478. doi: 10.1371/journal.pone.0233478 (PMC7721464; doi:10.1371/journal.pone.0233478)
Supplement: S1 Table — (DOCX) [file pone.0233478.s001.docx]

| S1 Table. Alleles proportions across survey years. | | | | | | |
| --- | --- | --- | --- | --- | --- | --- |
| Gene (Polymorphic codon) | **SNP** | **2010, n(%)** | **2011, n(%)** | **2012, n(%)** | **2013, n(%)** | **2014, n(%)** |
| *pfcrt* (72, 74, 75, 76) | C72 | 75 (100) | 73 (100) | 59 (100) | 70 (100) | 66 (100) |
|  | S72 | 0 (0.0) | 0 (0.0) | 0 (0.0) | 0 (0.0) | 0 (0.0) |
|  | M74 | 45 (60.0) | 42 (57.5) | 40 (67.8) | 56 (80.0) | 50 (75.8) |
|  | I74 | 27 (36.0) | 28 (38.4) | 19 (32.2) | 11 (15.7) | 15 (22.7) |
|  | M74/ I74 | 3 (4.0) | 3 (4.1) | 0 (0.0) | 3 (4.3) | 1 (1.5) |
|  | N75 | 45 (60.0) | 42 (57.5) | 40 (67.8) | 56 (80.0) | 50 (75.8) |
|  | E75 | 27 (36.0) | 28 (38.4) | 19 (32.2) | 11 (15.7) | 15 (22.7) |
|  | N75/ E75 | 3 (4.0) | 3 (4.1) | 0 (0.0) | 3 (4.3) | 1 (1.5) |
|  | K76 | 45 (60.0) | 42 (57.5) | 40 (67.8) | 56 (80.0) | 50 (75.8) |
|  | T76 | 27 (36.0) | 28 (38.4) | 19 (32.2) | 11 (15.7) | 15 (22.7) |
|  | K76/ T76 | 3 (4.0) | 3 (4.1) | 0 (0.0) | 3 (4.3) | 1 (1.5) |
|  |  | **N= 75** | **N = 73** | **N= 59** | **N= 70** | **N= 66** |
| *pfmdr1* (86, 184, 1246) | N86 | 48 (65.8) | 47 (60.2) | 59 (80.8) | 59 (78.7) | 54 (78.3) |
|  | Y86 | 25 (34.2) | 30 (38.5) | 14 (19.2) | 16 (21.3) | 15 (21.7) |
|  | N86/ Y86 | 0 (0.0) | 1 (1.3) | 0 (0.0) | 0 (0.0) | 0 (0.0) |
|  | Y184 | 7 (9.6) | 10 (12.8) | 10 (13.7) | 15 (20.0) | 20 (29.0) |
|  | F184 | 65 (89.0) | 63 (80.8) | 62 (84.9) | 54 (72.0) | 47 (68.1) |
|  | Y184/ F184 | 1 (1.4) | 5 (6.4) | 1 (1.4) | 6 (8.0) | 2 (2.9) |
|  | D1246 | 66 (90.4) | 74 (94.9) | 70 (95.9) | 72 (96.0) | 68 (98.6) |
|  | Y1246 | 7 (9.6) | 4 (5.1) | 3 (4.1) | 3 (4.0) | 1 (1.4) |
|  |  | **N = 73** | **N= 78** | **N = 73** | **N = 75** | **N = 69** |
| *pfdhfr* (51, 59, 108, 164) | N51 | 25 (31.3) | 20 (25.3) | 30 (44.1) | 34 (46.6) | 37 (53.6) |
|  | I51 | 52 (65.0) | 55 (69.6) | 37 (54.4) | 37 (50.7) | 30 (43.5) |
|  | N51/ I51 | 3 (3.7) | 4 (5.1) | 1 (1.5) | 2 (2.7) | 2 (2.9) |
|  | C59 | 16 (20.0) | 14 (17.7) | 28 (41.2) | 30 (41.1) | 34 (49.3) |
|  | R59 | 61 (76.3) | 61 (77.2) | 39 (57.3) | 41 (56.2) | 33 (47.8) |
|  | C59/ R59 | 3 (3.7) | 4 (5.1) | 1 (1.5) | 2 (2.7) | 2 (2.9) |
|  | S108 | 4 (5.0) | 4 (5.1) | 11 (16.2) | 6 (8.2) | 10 (14.5) |
|  | N108 | 76 (95.0) | 75 (94.9) | 57 (83.8) | 67 (91.8) | 59 (85.5) |
|  | I164 | 80 (100) | 79 (100) | 68 (100) | 73 (100.0) | 69 (100.0) |
|  | L164 | 0 (0.0) | 0 (0.0) | 0 (0.0) | 0 (0.0) | 0 (0.0) |
|  |  | **N= 80** | **N= 79** | **N= 68** | **N= 73** | **N= 69** |
| *pfdhps* (436, 437, 540, 581, 613) | S436 | 74 (93.7) | 69 (88.4) | 64 (92.8) | 71 (91.0) | 65 (90.3) |
|  | F436 | 5 (6.3) | 9 (11.6) | 5 (7.2) | 7 (9.0) | 7 (9.7) |
|  | A437 | 42 (53.2) | 36 (46.2) | 33 (47.8) | 40 (51.3) | 39 (54.2) |
|  | G437 | 37 (46.8) | 42 (53.8) | 36 (52.2) | 38 (48.7) | 33 (45.8) |
|  | K540 | 79 (100) | 78 (100) | 69 (100) | 78 (100) | 72 (100) |
|  | E540 | 0 (0.0) | 0 (0.0) | 0 (0.0) | 0 (0.0) | 0 (0.0) |
|  | A581 | 79 (100) | 77 (98.7) | 64 (92.7) | 74 (94.9) | 70 (97.2) |
|  | G581 | 0 (0.0) | 1 (1.3) | 5 (7.3) | 4 (5.1) | 2 (2.8) |
|  | A613 | 56 (70.9) | 68 (87.2) | 61 (88.4) | 66 (84.6) | 61 (84.7) |
|  | S613 | 23 (29.1) | 10 (12.8) | 8 (11.6) | 12 (15.4) | 11 (15.3) |
|  |  | **N= 79** | **N= 78** | **N= 69** | **N= 78** | **N= 72** |
| *pfk13* (580) | C580 | 70 (100) | 65 (100) | 56 (100) | 68 (100) | 73 (100) |
|  | Y580 | 0 (0.0) | 0 (0.0) | 0 (0.0) | 0 (0.0) | 0 (0.0) |
|  |  | **N= 70** | **N= 65** | **N= 56** | **N= 68** | **N= 73** |
