# Supplementary material for: Assessment of antimalarial drug resistant markers in asymptomatic Plasmodium falciparum infections after 4 years of indoor residual spraying in Northern Ghana
Source: PLoS One. 2020 Dec 7;15(12):e0233478. doi: 10.1371/journal.pone.0233478 (PMC7721464; doi:10.1371/journal.pone.0233478)
Supplement: S2 Table — (DOCX) [file pone.0233478.s002.docx]

| S2 Table. Multilocus *pfcrt, pfmdr1, pfdhfr and pfdhps* haplotypes across survey years | | | | | | | | | | | | | |
| --- | --- | --- | --- | --- | --- | --- | --- | --- | --- | --- | --- | --- | --- |
| Haplotype | **K76T** | **N86Y** | **N51I** | **C59R** | **S108N** | **A437G** | **K540E** | **Number of mutations** | **2010,**  **% (n/N)** | **2011,**  **% (n/N)** | **2012,**  **% (n/N)** | **2013,**  **% (n/N)** | **2014,**  **% (n/N)** |
| Wildtype | K | N | N | C | S | A | K | 0 | 0.0 (0/64) | 3.0 (2/66) | 3.7 (2/54) | 5.1 (3/59) | 3.8 (2/53) |
| Mutants | K | N | N | **C** | **N** | A | K | 1 | 4.7 (3/64) | 0.0 (0/66) | 1.9 (1/54) | 10.2 (6/59) | 18.9 (10/53) |
|  | K | N | N | C | S | **G** | K | 1 | 0.0 (0/64) | 0.0 (0/66) | 1.9 (1/54) | 0.0 (0/59) | 5.6 (3/53) |
|  | K | N | N | **R** | **N** | A | K | 2 | 1.6 (1/64) | 0.0 (0/66) | 1.9 (1/54) | 1.7 (1/59) | 1.9 (1/53) |
|  | K | **Y** | N | C | S | **G** | K | 2 | 1.6 (1/64) | 0.0 (0/66) | 0.0 (0/54) | 0.0 (0/59) | 0.0 (0/53) |
|  | **T** | N | N | C | **N** | A | K | 2 | 3.0 (2/64) | 3.0 (2/66) | 0.0 (0/54) | 3.4 (2/59) | 7.5 (4/53) |
|  | **T** | **Y** | N | C | S | A | K | 2 | 1.6 (1/64) | 0.0 (0/66) | 0.0 (0/54) | 0.0 (0/59) | 0.0 (0/53) |
|  | K | N | N | C | **N** | **G** | K | 2 | 0.0 (0/64) | 3.0 (2/66) | 11.0 (6/54) | 8.4 (5/59) | 5.6 (3/53) |
|  | K | **Y** | N | C | **N** | A | K | 2 | 0.0 (0/64) | 0.0 (0/66) | 0.0 (0/54) | 5.1 (3/59) | 0.0 (0/53) |
|  | K | N | **I** | **R** | **N** | A | K | 3 | 20.3 (13/64) | 19.7 (13/66) | 31.4 (17/54) | 22.0 (13/59) | 15.1 (8/53) |
|  | K | N | N | **R** | **N** | **G** | K | 3 | 6.3 (4/64) | 3.0 (2/66) | 1.9 (1/54) | 0.0 (0/59) | 1.9 (1/53) |
|  | K | **Y** | N | C | **N** | **G** | K | 3 | 3.0 (2/64) | 0.0 (0/66) | 1.9 (1/54) | 0.0 (0/59) | 1.9 (1/53) |
|  | **T** | N | N | C | **N** | **G** | K | 3 | 1.6 (1/64) | 1.5 (1/66) | 3.7 (2/54) | 3.4 (2/59) | 0.0 (0/53) |
|  | **T** | N | N | **R** | **N** | A | K | 3 | 3.0 (2/64) | 0.0 (0/66) | 0.0 (0/54) | 3.4 (2/59) | 0.0 (0/53) |
|  | K | **Y** | N | **R** | **N** | A | K | 3 | 0.0 (0/64) | 1.5 (1/66) | 0.0 (0/54) | 0.0 (0/59) | 0.0 (0/53) |
|  | **T** | **Y** | N | C | **N** | A | K | 3 | 0.0 (0/64) | 3.0 (2/66) | 0.0 (0/54) | 0.0 (0/59) | 0.0 (0/53) |
|  | **T** | **Y** | N | C | S | **G** | K | 3 | 0.0 (0/64) | 0.0 (0/66) | 1.9 (1/54) | 0.0 (0/59) | 1.9 (1/53) |
|  | K | N | **I** | **R** | S | **G** | K | 3 | 0.0 (0/64) | 0.0 (0/66) | 0.0 (0/54) | 1.7 (1/59) | 0.0 (0/53) |
|  | K | N | **I** | **R** | **N** | **G** | K | 4 | 10.9 (7/64) | 19.7 (13/66) | 11.0 (6/54) | 22.0 (13/59) | 22.6 (12/53) |
|  | K | **Y** | **I** | **R** | **N** | A | K | 4 | 6.3 (4/64) | 1.5 (1/66) | 3.7 (2/54) | 3.4 (2/59) | 0.0 (0/53) |
|  | **T** | N | **I** | **R** | **N** | A | K | 4 | 4.7 (3/64) | 7.6 (5/66) | 11.0 (6/54) | 0.0 (0/59) | 3.8 (2/53) |
|  | **T** | N | N | **R** | **N** | **G** | K | 4 | 1.6 (1/64) | 0.0 (0/66) | 0.0 (0/54) | 0.0 (0/59) | 0.0 (0/53) |
|  | **T** | **Y** | N | C | **N** | **G** | K | 4 | 4.7 (3/64) | 1.5 (1/66) | 1.9 (1/54) | 1.7 (1/59) | 0.0 (0/53) |
|  | **T** | **Y** | N | **R** | **N** | A | K | 4 | 1.6 (1/64) | 0.0 (0/66) | 0.0 (0/54) | 0.0 (0/59) | 0.0 (0/53) |
|  | K | **Y** | N | **R** | **N** | **G** | K | 4 | 0.0 (0/64) | 3.0 (2/66) | 0.0 (0/54) | 1.7 (1/59) | 1.9 (1/53) |
|  | K | **Y** | **I** | **R** | **N** | **G** | K | 5 | 4.7 (3/64) | 4.6 (3/66) | 1.9 (1/54) | 3.4 (2/59) | 1.9 (1/53) |
|  | **T** | N | **I** | **R** | **N** | **G** | K | 5 | 4.7 (3/64) | 4.6 (3/66) | 7.4 (4/54) | 3.4 (2/59) | 0.0 (0/53) |
|  | **T** | **Y** | **I** | **R** | **N** | A | K | 5 | 12.5 (8/64) | 10.7 (7/66) | 0.0 (0/54) | 0.0 (0/59) | 3.8 (2/53) |
|  | **T** | **Y** | N | **R** | **N** | **G** | K | 5 | 0.0 (0/64) | 1.5 (1/66) | 0.0 (0/54) | 0.0 (0/59) | 0.0 (0/53) |
|  | **T** | **Y** | **I** | **R** | **N** | **G** | K | 6 | 1.6 (1/64) | 7.6 (5/66) | 1.9 (1/54) | 0.0 (0/59) | 1.9 (1/53) |
| Note. Wild type amino acids are depicted in normal font, while mutated amino acids are in bold and underlined. | | | | | | | | | | | | | |
